# Anthocyanins from *Vitis coignetiae* Pulliat Inhibit Cancer Invasion and Epithelial-Mesenchymal Transition, but These Effects Can Be Attenuated by Tumor Necrosis Factor in Human Uterine Cervical Cancer HeLa Cells

**DOI:** 10.1155/2013/503043

**Published:** 2013-06-20

**Authors:** Jing Nan Lu, Won Sup Lee, Jeong Won Yun, Min Jeong Kim, Hye Jung Kim, Dong Chul Kim, Jae-Hoon Jeong, Yung Hyun Choi, Gon-Sup Kim, Chung Ho Ryu, Sung Chul Shin

**Affiliations:** ^1^Department of Internal Medicine, Institute of Health Sciences, Gyeongsang National University School of Medicine, 90 Chilam-dong, Jinju 660-702, Republic of Korea; ^2^The Fifth Hospital of Shijizhuang, Shijizhuang 050021, China; ^3^Department of Pharmacology, Institute of Health Sciences, Gyeongsang National University School of Medicine, Jinju 660-702, Republic of Korea; ^4^Department of Pathology, Institute of Health Sciences, Gyeongsang National University School of Medicine, Jinju 660-702, Republic of Korea; ^5^Division of Radiation Cancer Research, Korea Institute of Radiological and Medical Sciences, Seoul 660-702, Republic of Korea; ^6^Department of Biochemistry, Dongeui University College of Oriental Medicine and Department of Biomaterial Control (BK21 program), Dongeui University Graduate School, Busan 614-052, Republic of Korea; ^7^School of Veterinary Medicine, Gyeongsang National University, Jinju 660-701, Republic of Korea; ^8^Division of Applied Life Science (BK 21 Program), Institute of Agriculture and Life Science, Gyeongsang National University, Jinju 660-701, Republic of Korea; ^9^Department of Chemistry, Research Institute of Life Science, Gyeongsang National University, Jinju 660-701, Republic of Korea

## Abstract

Recently we have demonstrated that anthocyanins from fruits of *Vitis coignetiae* Pulliat (AIMs) have anticancer effects. Here, we investigate the effects of AIMs on cell proliferation and invasion as well as epithelial-mesenchymal transition (EMT) which have been linked to cancer metastasis in human uterine cervical cancer HeLa cells. AIMs inhibited the invasion of HeLa cells in a dose-dependent manner. AIMs inhibited MMP-9 expression in a dose-dependent manner. AIMs inhibited the motility of HeLa cells in a wound healing test. AIMs still suppressed NF-**κ**B activation induced by TNF. AIMs also inhibited EMT in HeLa cells. AIMs suppressed vimentin, N-cadherin, and *β*-catenin expression and induced E-cadherin. AIMs also suppressed expression of *β*-catenin and Snail, which was regulated by GSK-3. These effects of AIMs were also limited in the HeLa cells treated with TNF. In conclusion, this study indicates that AIMs have anticancer effects by suppressing NF-**κ**B-regulated genes and EMT, which relates to suppression of I**κ**B**α** phosphorylation and GSK-3 activity, respectively. However, the effects of AIMs were attenuated in the TNF-high condition.

## 1. Introduction

Most cancers usually follow a relatively orderly pattern in metastasis; it initially spreads to primary regional pelvic lymph nodes, then to farther regional nodes and distant sites. Eventually most of cancer patients die of metastatic lesions. Therefore, the control of metastasis is very important in the management of this disease. In the process of metastasis, tumor invasion is the first and essential step, which includes proteolytic digestion of the extracellular matrix (ECM) and cell migration through the basement membrane. Matrix metalloproteinase (MMPs), a family of zinc-dependent endopeptidases, play a critical role in ECM degradation, which is the starting point of cancer invasion. Among them, MMP-2 (gelatinase-A) and MMP-9 (gelatinase-B) are thought to be important in metastasis [1]. After this process, epithelial cancer cells need to change to metastasize other sites. Epithelial-mesenchymal transition (EMT) is a hypothesized program of development of epithelial cancer cells which lose epithelial characteristics and acquire invasive properties and stem cell-like features. The EMT is reported to be involved in cancer cell metastasis and dissemination [2]. 

Recently phytochemicals or food substances have been reported to safely show anticancer activity [3, 4] and to have anti-EMT properties [5]. Lots of efforts have been put into searching natural compounds with anti-cancer activities because modern anti-cancer drug development focuses on the agents that are less toxic to less compromise the patients' quality of life. 

Anthocyanins belong to a class of flavonoids. We isolated anthocyanins from *Vitis coignetiae* Pulliat (meoru in Republic of Korea) which has been used in Korean folk medicine for the treatment of inflammatory disorders and cancer. The intense dark red hue of this fruit is reflecting an abundance of anthocyanin pigments. We previously demonstrated the anticancer effects of AIMs in some cancers [6–8]. Despite the previous study, the target proteins involved in the metastasis which AIMs suppress remained inconclusive. Here, we investigated whether the anthocyanins isolated from meoru (AIMs) exert anticancer effects with special focus on invasion, EMT linked to metastasis.

## 2. Materials and Methods

### 2.1. Cell Culture and Chemicals

HeLa human uterine cervical cancer cells from the American Type Culture collection (Rockville, MD, USA) are cultured in RPMI 1640 medium (Invitrogen Corp, Carlsbad, CA, USA) supplemented with 10% (v/v) fetal bovine serum (FBS) (GIBCO BRL, Grand Island, NY, USA), 1 mM L-glutamine, 100 U/mL penicillin, and 100 *μ*g/mL streptomycin at 37°C in a humidified atmosphere of 95% air and 5% CO_2_ incubator. Molecular mass markers for proteins were obtained from Pharmacia Biotech (Saclay, France). Antibodies against COX-2, cyclin D1, c-Myc, cIAP-1, cIAP-2, XIAP, Bcl-2, Bcl-xL, MMP-2, MMP-9, VEGF, ICAM-1, E-Cadherin, and β-catenin were purchased from Santa Cruz Biotechnology Inc. (Santa Cruz, CA, USA). Antibodies against phospho-IKB-*α* (Ser 32/36), IKB, Vimentin, Snail, N-Cadherin, phospho-GSK3 β (Ser 9) were purchased from Cell signaling Technology, Inc. (Beverly, MA, USA). Antibody against β-actin was from Sigma (Beverly, MA, USA). Peroxidase-labeled donkey anti-rabbit and sheep anti-mouse immunoglobulin and an enhanced chemiluminescence (ECL) kit were purchased from Amersham (Arlington Heights, IL, USA). All other chemicals not specifically cited here were purchased from Sigma Chemical Co. (St. Louis, MO, USA).

### 2.2. Preparation of Anthocyanins

Anthocyanins were isolated as previously described [6]. The composition of anthocyanidins isolated from meoru (AIMs) was as follows: delphinidin-3,5-diglucoside : cyanidin-3,5-diglucoside : petunidin-3,5-diglucoside : delphinidin-3-glucoside : malvdin-3,5-diglucoside : peonidin-3,5-diglucoside : cyanidin-3-glucoside : petunidin-3-glucoside : peonidin-3-glucoside : malvidin-3-glucoside = 22.76 : 53.12 : 6.97 : 0.84 : 1.86 : 1.86 : 5.65 : 1.05 : 4.82 : 1.05.

### 2.3. Cell Proliferation Assays

For the cell viability assay, the cells were seeded onto 24-well plates at a concentration of 5 × 10^4^ cells/mL and treated with AIMs for 48 h, and the number of surviving cells was counted using trypan blue exclusion methods. 

### 2.4. Wound Healing Assay

The wound healing assays were conducted according to the methods described previously. HeLa cells were grown on 35 mm dish plate to 100% confluent monolayer and then scratched to form a 100 *μ*m “wound” using sterile pipette tips. The cells were then cultured in the presence or absence of AIMs (400 *μ*g/mL) in serum-free media for 24 h. The images were recorded at 12 h and 24 h after the scratch using an Olympus photomicroscope.

### 2.5. Cell Invasion Assay

 For the cell invasion assays, the cells were cultured in serum-free media overnight. Cells (5 × 10^4^ cells) were loaded onto precoated Matrigel 24-well invasion chambers (BD Biosciences, San Jose, CA, USA) in the presence or absence of AIMs (400 *μ*g/mL). Then 0.5 mL of 5% fetal calf serum medium was added to the wells of the plate to serve as the chemoattractant. The Matrigel invasion chambers were incubated for 24 h. Invading cells were fixed with 10% formalin, stained with DAPI, and counted.

### 2.6. Gelatin Zymography

The gelatinolytic activities for secreted MMP-2 and MMP-9 in the culture medium were assayed by electrophoresis on 8% polyacrylamide gels containing 1 mg/mL gelatin at 4°C. Mix the culture media (15 *μ*L) with 15 *μ*L Tris-Glycine SDS Sample Buffer (2x), and let them stand for 10 minutes at room temperature. The mixture (20 *μ*L) was loaded on the polyacrylamide gels, which were run at 120 V, washed in 2.5% Triton X-100 for 1 h, and then incubated for 16 h at 37°C in activation buffer (50 mM Tris-HCl, pH 7.5, and 10 mM CaCl_2_). After staining with Coomassie blue (10% glacial acetic acid, 30% methanol, and 1.5% Coomassie brilliant blue) for 2-3 h, the gel was washed with a solution of 10% glacial acetic acid and 30% methanol without Coomassie blue for 1 h. White lysis zones indicating gelatin degradation were revealed by staining with Coomassie brilliant blue.

### 2.7. Western Blotting

 Total cell lysates were obtained using lysis buffer containing 0.5% SDS, 1% NP-40, 1% sodium deoxycholate, 150 mM NaCl, 50 mM Tris-Cl (pH 7.5), and protease inhibitors. The concentrations of cell lysate proteins were determined by means of the Bradford protein assay (Biorad lab, Richmond, CA, USA) using bovine serum albumin as the standard. Molecular mass markers for proteins were obtained from Pharmacia Biotech (Saclay, France). For Western blotting, 30 micrograms of proteins were resolved by electrophoresis, electrotransferred to polyvinylidene difluoride membranes (Millipore, Bedford, MA, USA), and then incubated with primary antibodies followed by a secondary antibody conjugated to peroxidase. Blots were developed with an ECL detection system.

### 2.8. Immunocytochemistry

 The cells were placed on coverslips coated with poly-L-lysine (1 mg/mL) in 6-well plates. They were fixed in 4% paraformaldehyde for 10 min followed by 1.0% H_2_O_2_/0.1 M PBS treatment for 30 min after washing twice in phosphate-buffered saline (PBS). Then, 0.3% Triton/0.1 M PBS was treated for 5 min and then washed twice in buffered saline. They were incubated in 5% serum solution for 30 min at room temperature and then the serum solution was removed with suction. They were incubated in buffered saline with a 1 : 50 dilution of primary antibodies for p65 NF-*κ*B (Santa Cruz Biotechnology, Santa Cruz, CA, USA) for 2 h and then washed in buffered saline three times for 10 minutes each at room temperature. They were incubated in buffered saline with a 1 : 250 dilution of biotinylated secondary antibodies (Vector Elite Kit). Positive staining was visualized with diaminobenzidine, followed by a light hematoxylin counter staining.

### 2.9. Statistical Analysis

Each experiment was performed in triplicate. The results were expressed as means ± SD. Significant differences were determined using the one-way analysis of variance (ANOVA) with posttest Newman-Keuls in the cases of at least three treatment groups and Student's *t* test for two-group comparison. Statistical significance was defined as *P* < 0.05.

## 3. Results

### 3.1. AIMs Suppress the Proliferation and Invasion of HeLa Cells

**Figure 1:**
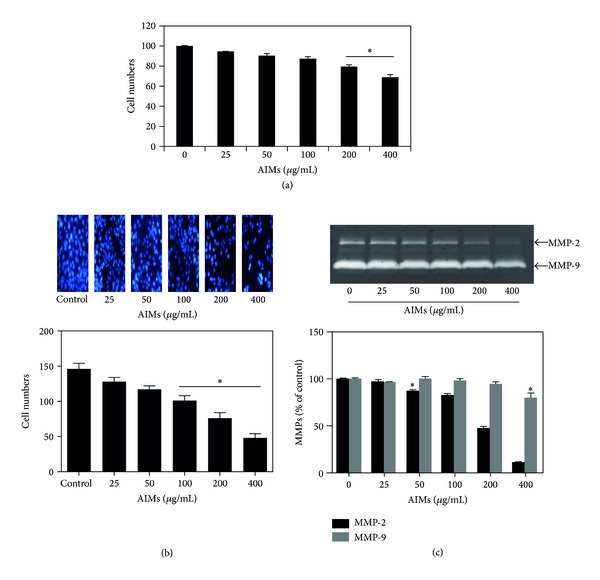
Dose-dependent inhibitory effects of AIMs on HeLa cell proliferation and invasion. (a) Growth inhibition of HeLa cells. Cells were seeded at 5 × 10^4^ cells/mL and treated with AIMs for 24 h with the indicated concentrations. (b) Effects on invasion of HeLa cells. The cells (5 × 10^4^ cells) were loaded on precoated Matrigel 24-well invasion chambers (BD Biosciences) in the presence or absence of AIMs (25–400 *μ*g/mL). HeLa cells were treated with AIMs for 18 hr in a Matrigel-coated transwell. (c) MMP-2 and MMP-9 protein levels were measured by gelatin zymography. Cells were incubated for 48 h without or with AIMs (25–400 *μ*g/mL). Values represent means ± SD from three independent experiments. *P < 0.05 versus control.

At first, the growth of cancer cells was assessed by trypan blue exclusion methods. The assay revealed that the growth of HeLa cells was inhibited by AIMs in a dose-dependent manner (Figure 1(a)). Cancer cell invasion is the first step in cancer metastasis. Therefore, we tested the effects of AIMs on cell invasion. AIMs markedly inhibited HeLa cell invasion in a dose-dependent manner as measured by Matrigel invasion assays (Figure 1(b)). Invasion process includes proteolytic digestion of the extracellular matrix (ECM), and MMPs are involved in degrading all kinds of extracellular matrix proteins [9, 10]. In particular, the secreted MMP-2 and MMP-9 are important molecules in cancer cell invasion [9]. To investigate the molecular mechanisms, we also measured the gelatinolytic activities of the secreted MMP-2 and MMP-9 in the culture media with gelatin zymographic analyses. As indicated in Figure 1(c), AIMs have markedly suppressed the gelatinolytic activities of secreted MMP-9 in dose-dependent manner, compared to the suppressive effects of AIMs on MMP-2. These findings suggest that AIMs suppress the invasion predominantly through MMP-9 suppression.

### 3.2. TNF Attenuated the Inhibitory Effects of AIMs on Cell Migration and Invasion in HeLa Cells by Upregulating of MMP-2

**Figure 2:**
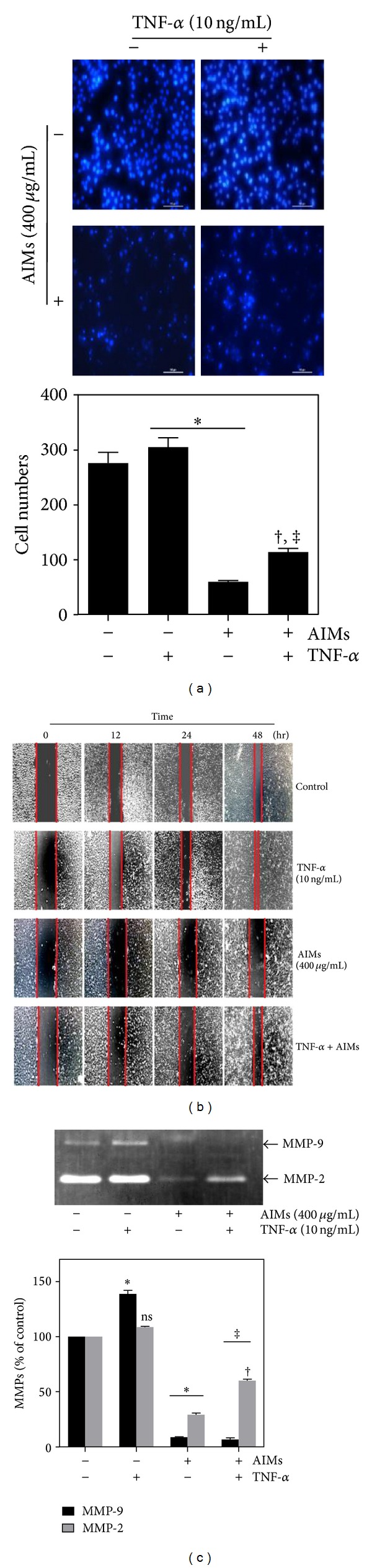
Effects of AIMs on cancer cell migration and invasion in HeLa cells treated with TNF. (a) Cells were serum-starved for 24 hrs with or without AIMs (400 *μ*g/mL). Cells (5 × 10^4^ cells) were loaded onto precoated Matrigel 24-well invasion chambers in the presence or absence of TNF (10 ng/mL). The Matrigel invasion chambers were incubated for 24 h. (×400; the length of scale bar, 40 *μ*m). (b) Cells were grown to 100% confluency on 30 mm cell culture dishes coated with collagen and then serum-starved for 24 hrs with or without TNF (10 ng/mL) and/or AIMs (400 *μ*g/mL) for 24 h. A linear scratch was made on the culture dish through the cell layer using a pipette tip. After washing with PBS, serum-free media with or without AIMs were added. Photographs of the etched area in a Petri dish were taken at the interval of 0 h, 12 h, 24 h, and 48 h after the scratch to evaluate cell movement into the wounded area. Data were representative of three independent experiments. (c) MMP-2 and MMP-9 protein levels were measured by gelatin zymography. Cells were incubated for 48 h without or with AIMs (25–400 *μ*g/mL). Values represent means ± SD from three independent experiments. *P < 0.05 versus control, ^†^ *P* < 0.05 versus AIM alone.

MMP-9 expression is regulated by NF-*κ*B [11]. TNF is a well-known NF-*κ*B stimulator. Actually, TNF binds two receptors, TNF receptor 1 (TNF-R1) and TNF receptor 2 (TNF-R2). With TNF binding to TNF-R, three pathways can be initiated: NF-*κ*B, MAPK, and death signaling. The first one is involved in cell survival pathway and the others in proapoptotic or death pathway [12]. Since the capability of TNF-induced cell death is weak and often masked by the antiapoptotic effects of NF-*κ*B in most of epithelial cancers, TNF actually augments invasion and induces EMT cells rather than induces cell death. Here, we use TNF to clearly demonstrate the effects of AIMs on invasion and EMT as well as NF-*κ*B. Unexpectedly, TNF diminished the antiinvasive effect of AIMs (Figures 2(a) and 2(b)). Likewise, AIMs significantly inhibited cancer cell migration in the wound healing test, but the antimigratory effect of AIMs was also diminished by TNF (Figure 2(b)). To confirm the effects of AIMs at the molecular level, we measured the gelatinolytic activities of the secreted MMP-2 and MMP-9 by gelatin zymographic analyses. As indicated in Figure 2(c), AIMs have markedly suppressed the secreted MMP-9 even in the TNF-treated cells. TNF increased MMP-2 in the AIM-treated cells. This finding suggests that the inhibitory effects of AIMs are related to the suppression of MMP-9 and that TNF can attenuate the cancer invasion and migration by the induction of MMP-2 expression. 

### 3.3. AIMs Suppressed NF-*κ*B Activity through Suppression of I*κ*B*α* Phosphorylation

**Figure 3:**
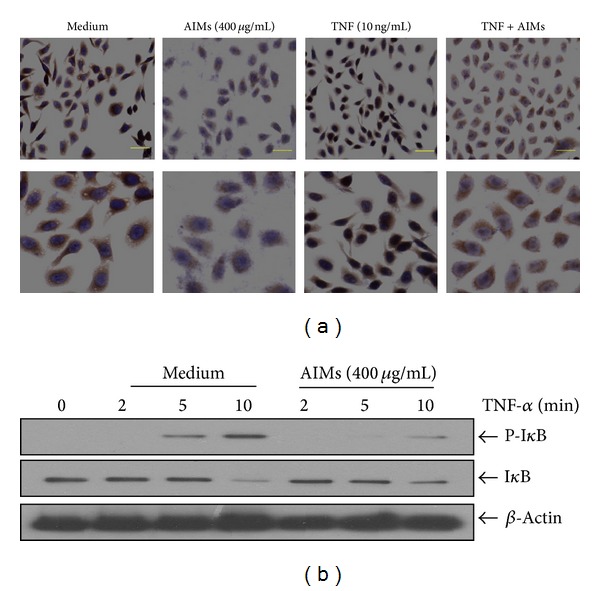
Effects of AIMs on NF-*κ*B activity and I*κ*B*α* phosphorylation. (a) Immunocytochemical analysis of NF-*κ*B (p65) localization in HeLa cells. Cells were pretreated with AIMs (400 *μ*g/mL) or 0.1% DMSO (vehicle control) for 24 h and then treated with TNF (10 ng/mL) for 30 min (×400; the length of scale bar, 50 *μ*m). (b) Cells were pretreated with AIMs (400 *μ*g/mL) for 1 h and then treated with TNF (10 ng/mL) for the indicated times. Data were representative of two independent experiments.

NF-*κ*B pathway is involved in cancer cell proliferation, invasion, and metastasis [13, 14]. MMP-9 expression is preferentially regulated by NF-*κ*B, but MMP-2 is also regulated by NF-*κ*B [11]. Here, we reconfirmed the inhibitory effects of AIMs on TNF-induced NF-*κ*B activation which had been reported in hepatocellular carcinoma cells [7]. Here, we reinvestigated whether AIMs inhibit NF-*κ*B activation using immunohistocytochemistry. The merit of immunohistochemistry is easy in showing NF-*κ*B (p65) translocation into the nucleus which means activation. As we expected, TNF enhanced the NF-*κ*B translocation into the nucleus and AIMs inhibited TNF-induced NF-*κ*B activation (Figure 3(a)). NF-*κ*B activation is known to require the degradation of I*κ*B*α* through phosphorylation by kinases. Next, we tested whether AIMs suppressed TNF-induced phosphorylation of I*κ*B*α*. The degradation of I*κ*B*α* through phosphorylation was seen as early as 5 min after adding TNF, and AIMs suppressed the TNF-induced phosphorylation of I*κ*B*α* in HeLa cells, but the inhibitory strength was strong enough to completely prevent I*κ*B*α* phosphrylation induced by TNF (Figure 3(b)). This finding suggests that AIMs inhibit NF-*κ*B activity through suppression of I*κ*B*α* phosphorylation and that the inhibitory effects of AIMs on NF-*κ*B are limited in TNF high conditions. 

### 3.4. AIMs Suppressed NF-*κ*B-Regulated Proteins Involved in Cancer Metastasis, Even in TNF-Treated Condition

**Figure 4:**
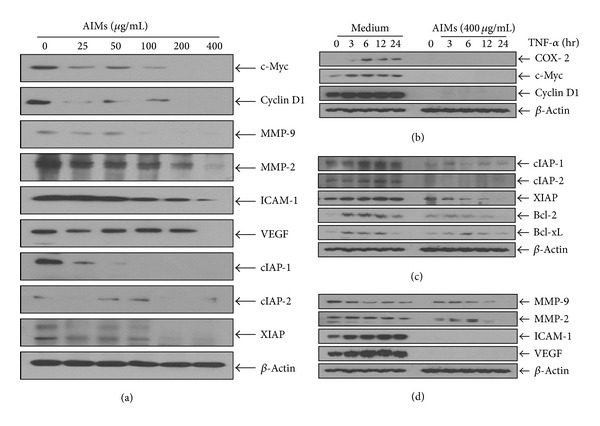
Effects of AIMs on NF-*κ*B-regulated proteins involved in cancer metastasis. (a) Cells (5 × 10^4^ cells) were either left untreated or pretreated with AIMs (25–400 *μ*g/mL) for 24 h. (b)–(d) Cells (5 × 10^4^ cells) were either left untreated or pretreated with AIMs (400 *μ*g/mL) for 24 h and then were exposed to TNF (10 nM) for indicated times. Whole-cell extracts were prepared, and 30 *μ*g of the whole-cell lysate was analyzed by Western blot using antibodies against various NF-*κ*B-regulated proteins involved in (b) the cancer cell proliferation, (c) anti-apoptosis, and (d) invasion (MMP-2 and MMP-9), adhesion (ICAM-1), and angiogenesis (VEGF). Data were representative of two independent experiments.

To confirm the AIMs effects on NF-*κ*B, we investigated the effects of AIMs on NF-*κ*B-regulated proteins involved in cancer metastasis. Western blot analyses revealed that AIMs suppressed the NF-*κ*B-regulated gene expressions of which basal level was high (Figure 4(a)). These findings suggest that AIMs suppress NF-*κ*B-regulated gene expressions linked with cancer proliferation, invasion, adhesion, and angiogenesis.

Next we observed the TNF effects on NF-*κ*B-regulated proteins (COX-2, Cyclin D1, and c-Myc) involved in cancer cell proliferation. TNF stimulated the expression of these genes, and pretreatment with AIMs completely prevents the activation of these genes of proliferation (Figure 4(b)). TNF-induced cell death is often masked because of the activation of the antiapoptotic effects of NF-*κ*B. Next we tested the effects of AIMs on antiapoptotic proteins (XIAP, IAP1, IAP2, Bcl-2, and Bcl-xL). TNF significantly activated these genes. Although pretreatment with AIMs significantly suppressed the expression of XIAP and Bcl-2 induced by TNF, the inhibitory effects of AIMs were not significant on IAP1, IAP2, and Bcl-xL (Figure 4(c)). Next we tested regarding molecules involved in invasion, adhesion, and angiogenesis (MMP-2, MMP-9, ICAM-1, and VEGF). TNF significantly activated these genes, and the treatment with AIMs significantly prevented the TNT-induced activation of these genes (Figure 4(d)). The inhibitory effects of AIMs on NF-*κ*B-regulated gene expressions linked with anti-apoptosis and invasion were somewhat attenuated by TNF. These findings suggest that pretreatment with AIMs can prevent TNF-stimulated genes involved in cell proliferation, cell adhesion, and angiogenesis, but not completely, especially those involved in cell invasion and anti-apoptosis in TNF-treated condition although AIMs significantly suppress the genes involved in cancer cell anti-apoptosis and invasion in TNF-untreated condition. 

### 3.5. AIMs Suppressed TNF-Induced Cell Elongation

**Figure 5:**
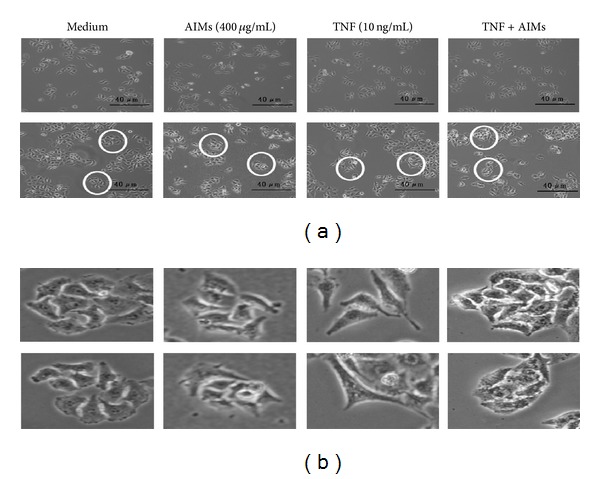
TNF-induced effects on changes in cell morphology of HeLa cells. (a) Cells seeded at 5 × 10^4^ cells/mL were pretreated with AIMs (400 *μ*g/mL) for 1 h and then treated with TNF (10 ng/mL) for 24 hr, and we observed the morphologic changes under the microscope. (×200; the length of scale bar, 40 *μ*m). (b) Magnifying pictures for the area marked with circles. HeLa cells become elongated and spindle shaped after TNF treatment, and AIMs prevent the morphologic changes. Data were representative of three independent experiments.

Next, we investigated the effects of AIMs on EMT. As shown in Figure 5, TNF induced significant cell morphology changes; larger proportion of cancer cells become elongated and spindle shaped after TNF treatment. AIMs suppressed the elongation of HeLa cells induced by TNF. These results suggest that TNF may trigger EMT and that AIMs may suppress the effects of TNF on EMT of HeLa cells.

### 3.6. AIMs Suppressed EMT and Prevented TNF-Induced EMT in HeLa Cells

**Figure 6:**
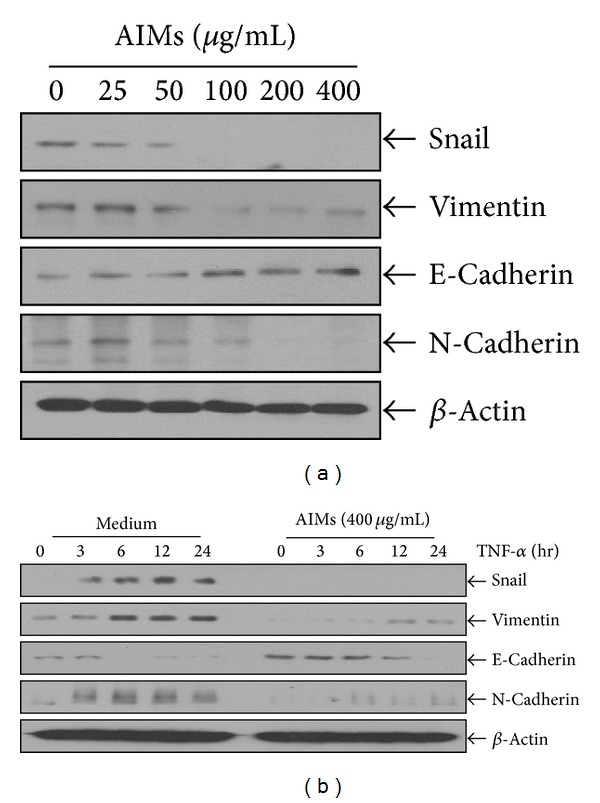
Effects of AIMs on EMT involved in cancer metastasis. (a) Cells (5 × 10^4^ cells) were either left untreated or pretreated with AIMs (25–400 *μ*g/mL) for 24 h. (b) Cells (5 × 10^4^ cells) were either left untreated or pretreated with AIMs (400 *μ*g/mL) for 24 h and then were exposed to TNF (10 nM) for indicated times. Whole-cell extracts were prepared, and 30 *μ*g of the whole-cell lysate was analyzed by Western blot. Data were representative of two independent experiments.

EMT is reported to be involved in cancer cell metastasis [2]. To confirm the inhibitory effects of AIMs on EMT, we investigated molecular changes regarding EMT after treatment with TNF and AIMs. Western blot revealed that AIMs suppressed mesenchymal markers such as Snail, vimentin, and N-cadherin and induced E-cadherin which is an epithelial marker (Figure 6(a)). EMT can be triggered by TNF. Here, we also investigated whether AIMs can prevent TNF-induced EMT in HeLa cells. As shown in Figure 6(b), TNF induced mesenchymal markers (Snail, vimentin, and N-cadherin) and suppressed an epithelial marker (E-cadherin), suggesting that TNF induced EMT of the cancer cells. These results indicated that AIMs could suppress EMT and prevent TNF-induced EMT. 

### 3.7. AIMs Inhibited β-Catenin and Phosphorylation of Serine-9 in Glycogen Synthase Kinase-3β (GSK-3β)

**Figure 7:**
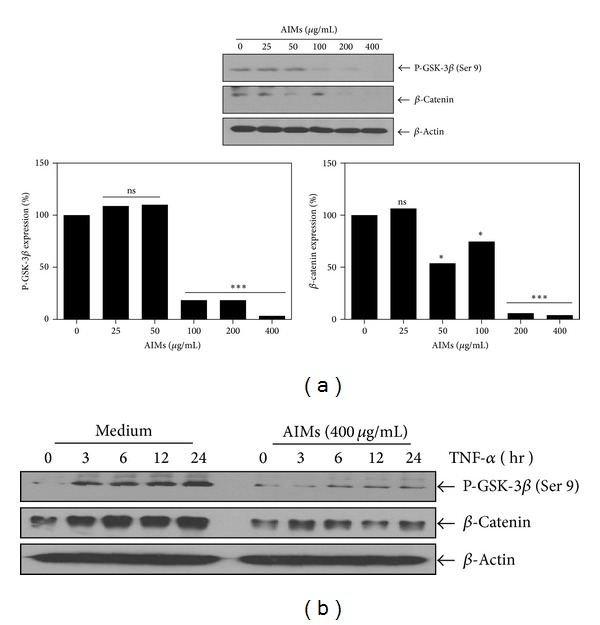
Effects of AIMs on β-catenin and phosphorylation of serine-9 in glycogen synthase kinase-3β (GSK-3β). (a) Cells (5 × 10^4^ cells) were either left untreated or pretreated with AIMs (25–400 *μ*g/mL) for 24 h. (b) Cells (5 × 10^4^ cells) were either left untreated or pretreated with AIMs (400 *μ*g/mL) for 24 h and then were exposed to TNF (10 nM) for indicated times. Whole-cell extracts were prepared, and 30 *μ*g of the whole-cell lysate was analyzed by Western blot. Data were representative of two independent experiments.

The major function of Snail is known for the induction of EMT, and Snail is phosphorylated by glycogen synthase kinase-3 (GSK-3) which is encoded by two known genes, GSK-3*α* and GSK-3β. The enzymatic activity is regulated by phosphorylation of certain GSK-3 residues. Phosphorylation at tyrosine-219 in GSK-3*α* or tyrosine-216 in GSK-3β enhances the activity of GSK-3, while phosphorylation of serine-21 in GSK-3*α* or serine-9 in GSK-3β significantly decreases the activity [15]. In addition, the canonical Wnt signalling pathway has a dominant role in regulating EMT in cancer as well as morphogenesis [16, 17]. When the canonical Wnt signal is activated, it leads to a change in the amount of β-catenin [18]. GSK-3 is also involved in phosphorylation of β-catenin which leads to the destruction of β-catenin. To investigate the mechanism responsible for anti-EMT effects of AIMs, we assessed the changes in β-catenin and phosphorylated GSK3β after treatment with AIMs. Here we assessed phosphorylation of serine-9 in GSK-3β. Western blot revealed that AIM suppressed β-catenin and phosphorylated GSK-3β (Ser 9) in a time-dependent manner (Figure 7(a)). These findings suggested that AIMs should suppress EMT at least in part by suppressing β-catenin and augmenting GSK-3 activity. Here, we confirmed this finding in HeLa cells treated with TNF. As shown in Figure 7(b), TNF induced phosphorylation of GSK-3β (Ser 9) and expression of β-catenin, and AIMs suppressed the TNF-effects on GSK-3β (Ser 9) and β-catenin. These results indicated that AIMs should prevent TNF-induced EMT at least in part by suppressing β-catenin and augmenting GSK-3 activity. 

## 4. Discussion

This study was designed to investigate the anticancer effects of AIMs and their underlying mechanisms with special focus on invasion and EMT relating to metastasis in HeLa human uterine cervical cancer cells. We found that AIMs significantly attenuated the cancer cell invasion, which was through the inhibition of MMP-9 expression and EMT. The potency of inhibitory effects of AIMs on migration and MMP-2/MMP-9 was increased by starvation; the effects of AIMs shown in Figure 2 were more potent than those in Figure 1 regarding migration and MMP-2/MMP-9 expression. These synergistic effects between anticancer drug and starvation were reported [19]. The compensatory increase in MMP-2 after TNF treatment can also be related to TNF-induced activated p38 MAPK activity [20] and the property of AIMs that increases p38 MAPK activity [8]. 

MMP-9 expression is one of the EMT markers and preferentially regulated by NF-*κ*B [11]. Therefore, we determined whether AIMs inhibited NF-03BAB-regulated proteins associated with cancer metastasis; we found that AIMs inhibited the proteins involved in cell proliferation, anti-apoptosis, adhesion, and angiogenesis in the TNF-untreated cells. All of these proteins are important in cancer metastasis. COX-2 and Cyclin D1 are overexpressed in a variety of cancers and mediate cancer cell proliferation [21], and c-Myc is also involved in cancer cell proliferation [22]. Many cancer cell lines are resistant to TNF-induced cell death because NF-*κ*B enhances the transcription of antiapoptotic proteins that interfere with death signaling [13], and TNF indeed augments cell proliferation in some of the cancer cells [13, 22]. In this study TNF also augmented all of these proteins involved in cell proliferation. The roles of MMP-2, MMP-9, ICAM-1, and VEGF in invasion, adhesion, and angiogenesis of cancer are well known and regulated by NF-*κ*B [14]. Previous studies showed anthocyanins suppressed NF-*κ*B activities [22–24]. Here, we found that TNF diminished the inhibitory effects of AIMs on NF-*κ*B. This is a little bit different from our previous result demonstrating that AIMs completely suppressed TNF-induced NF-*κ*B activation and I*κ*B*α* phosphorylation in Hep3B hepatocellular carcinoma cell line [6]. On the basis of these two results, the inhibitory effects of AIMs on NF-*κ*B appear to be correlated with that on the I*κ*B*α* phosphorylation in that the inhibitory effect of AIMs on NF-*κ*B is limited in TNF-treated HeLa cells where AIMs incompletely inhibited the I*κ*B*α* phosphorylation whereas AIMs completely inhibited the NF-*κ*B activity even in TNF-treated Hep3B cells where AIMs inhibit completely inhibited I*κ*B*α* phosphorylation [6]. The potency of anticancer effect of AIMs may vary depending on the cell types. 

We also found that TNF induced EMT and AIMs suppressed EMT. EMT that occurs during embryonic development has begun to attract attention as a potential mechanism for tumor cell metastasis. Snail is a well-known transcription factor that promotes EMT by repressing E-cadherin expression. Snail is known to be degraded and exported from the nucleus by GSK-3β [25]. Here we demonstrated that AIMs suppressed Snail expression and induced E-cadherin expression. The previous studies report that E-cadherin was not expressed in HeLa cells but actually downregulated by methylation at the promoter site [5], and the band can be seen and upregulated by a certain treatment [26]. In addition, AIMs enhanced GSK-3β activity by suppressing phosphorylation of GSK-3β (Ser 9). GSK-3 is also linked to pathways of cell proliferation and apoptosis. GSK-3 phosphorylates β-catenin, thus resulting in its degradation [15]. GSK-3 is also a part of the canonical β-catenin/Wnt pathway involved in cancer proliferation and EMT. In this study, we found that AIMs suppressed p-GSK3β (Ser 9) and β-catenin. There is only one study reported regarding the effects of pure anthocyanins on GSK-3. It revealed that cyanidin-3-glucoside upregulated p-GSK3β (Ser 9) in a rat neuroblastoma cell line [27]. This result is opposite to ours. However, we confirmed this finding in the cells treated with TNF, and there is one study that shows that the extract of skin of muscadine grape contains a predominant anthocyanin suppressed p-GSK3β (Ser 9) [28], which is supporting our results. There is no report available regarding the direct effects of anthocyanins on β-catenin except one study that demonstrated that food containing anthocyanins (white currant, a kind of berries) suppressed β-catenin [29]. In that context, the results from this study are significant as we report for the first time that anthocyanins inhibit EMT by suppressing p-GSK3β (Ser 9).

The limitation of this study is as follows. Firstly, we used TNF to clearly demonstrate the inhibitory effects on the genes involved in cancer cell survival, proliferation, invasion, EMT, and angiogenesis. TNF is usually increased in patients with advanced cancers [30, 31]. In addition, TNF is closely related to cancer progression and the patients' quality of life [30, 31]. Therefore, the pathophysiological relevance of TNF-*α*-induced NF-*κ*B activation is underlined in cancer study. Here, by using TNF we can confirm the effects of AIMs on HeLa cells. Furthermore TNF is abundantly released in *in vivo* situation like chronic inflammatory disorders, as well as cancer, and a TNF inhibitor is actually useful for the control of inflammatory disorders [32]. Therefore, TNF is a good therapeutic target for treatment of inflammatory disorder as well as cancer to reduce proinflammatory response. 

Secondly, regarding the anti-EMT effects of AIMs, since E-cadhrin in HeLa cells is downregulated by promoter methylation and phytochemicals have properties of demethylation, there is another possibility that demethylation could be a mechanism. Unfortunately, here we did not investigate the further detailed mechanisms. We tested another cell line (A549 cells); the results were of the same pattern (data not shown). In this study, we also found that AIMs did not prevent completely either TNF-driven EMT or TNF-driven MMP induction, suggesting in TNF-high condition, the efficacy might be limited in HeLa cell-like cancer cells.

Lastly, the concentration of AIMs in this study seems to be high, and we did not show whether this concentration is attainable *in vivo*, but the concentration of AIMs used in the present study is consistent with those in many other studies on the antitumor effect of anthocyanins [27, 33].

**Figure 8:**
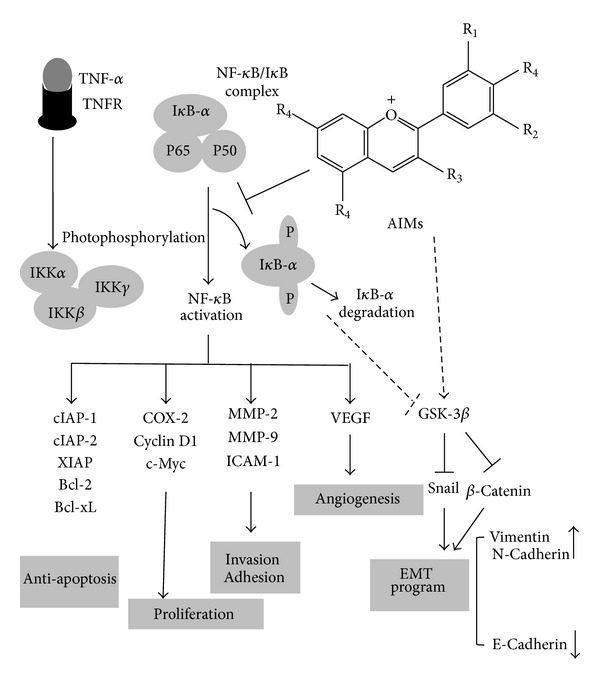
Schematic representation on anticancer effects of AIMs on HeLa human uterine cervical cancer cells. AIMs suppressed invasive effects of HeLa cells by suppression of MMP-9 by suppression of NF-*κ*B through at least inhibiting I*κ*B phosphorylation. AIMs clearly suppressed NF-*κ*B activation and the expression of NF-*κ*B-regulated proteins by inhibiting I*κ*B phosphorylation and prevented EMT by suppression of Snail and β-catenin through at least in part augmenting GSK-3 activity. Here, TNF participated in induction of NF-*κ*B-regulated proteins involved in cancer cell proliferation (cyclin D1 and COX-2), anti-apoptosis (XIAP, IAP1, IAP2, and Bcl-xL), and invasion and angiogenesis (MMP-2, MMP-9, ICAM-1, and VEGF) and also induced EMT. Taken together, this study suggested that suppression of NF-*κ*B and GSK-3β is an important factor for anticancer effects on cancer invasion effects as well as other metastatic effects and EMT in HeLa cells, respectively.

In summary, this study suggests that the anthocyanins isolated from meoru (AIMs) should exert anticancer effects by suppressing NF-*κ*B-regulated genes involved in cancer cell proliferation, anti-apoptosis, invasion, adhesion, and angiogenesis through inhibition of I*κ*B*α* phosphorylation, and by suppressing EMT through inhibition of Snail and β-catenin GSK-3 activity (Figure 8). However, the anticancer activities of AIMs were attenuated by TNF treatment, which suggests that the therapeutic efficacy of AIMs, if AIMs or meoru should apply to cancer patients, might be limited in the TNF high situation like the patients with far advanced cancer and cachexia. This study provides evidence that AIMs might have anticancer effects on human uterine cervical cancer.
